# Balancing the Demands of Older People and Care Services of Healthy Aging: Assessment and Positioning of Care Facilities

**DOI:** 10.3389/ijph.2025.1607249

**Published:** 2025-06-30

**Authors:** Kai Zhang, Dan Li, Xiaoting Cheng

**Affiliations:** ^1^ School of Public Administration, Sichuan University, Chengdu, Sichuan, China; ^2^ Business School, Sichuan University, Chengdu, Sichuan, China

**Keywords:** demands, older people, care services, healthy aging, recommendation methods, fuzzy-set qualitative comparative analysis

## Abstract

**Objectives:**

This study aims to analyze the demands of older people for care services and to evaluate and position care facilities to accommodate the requirements of healthy aging.

**Methods:**

Data on the demand for care services in Chengdu were collected. Fuzzy-set qualitative comparison analysis was used for demands analysis. Probabilistic linguistic term sets were used to assess and position facilities. Finally, the recommendations methods were provided by different demands of older people.

**Results:**

There were four paths to produce the high demands for care services of older people. Among the six types of services, medical services, psychological counseling, and nursing services had the greatest impact, while diet conditions had the least. The older people with clear demands can select facilities by the first recommendation method, while those with unclear demands can choose from four types of facilities: recommended, non-recommended, suitable for forward-thinking customers, and suitable for concerned customers.

**Conclusion:**

This study provides valuable insights into older people, care facilities, and governments. The older people can choose suitable facilities by their demands, while facilities can enhance service quality accordingly. Governments can allocate resources efficiently and promote healthy aging.

## Introduction

The aging population phenomenon and the concomitant decline in birth rates have led to an escalation in the aging process across virtually all nations [1]. Furthermore, countries across the globe actively promote and implement policies and protocols to address the challenges posed by the aging population [2, 3]. At the end of 2022, China’s population aged 60 and over was about 280 million, accounting for 19.8% of the total population. This number is expected to reach 400 million by 2035. Meanwhile, as the number of older people increases, the demands for healthcare services are also rising rapidly [4], which provides new challenges and opportunities for care facilities [5, 6]. Despite the Chinese cultural emphasis on filial piety and home-based care, evidence suggests that family support systems are increasingly inadequate for the care of older people in China [7]. As a result, care facilities are necessary as places of professional care for older people with chronic diseases, disabilities, and other problems. It is essential for care facilities to meet the demands for care services [8]. Therefore, balancing the supply and demands of care service for older people has become the key to promoting the development of the care service system.

However, the growing diversity of older people’s care demands, coupled with a decline in the service quality of care facilities, has led to an imbalance between the supply of and demand for healthcare for older people [9, 10]. Effectively addressing this issue requires a comprehensive understanding of older people’s specific demands and preferences. Previous studies have explored the factors influencing older people’s care demands, with surveys conducted in various regions across nations [11–14]. These studies have consistently demonstrated that care service demands are influenced by factors such as gender, age, education, occupation, economic status, and activities of daily living [15–19]. Furthermore, previous research has examined spatial planning for care facilities, the requirements for in-home care, and the impact of healthcare services on care demands [20–23].

In developing countries like China, care services for older people have increasingly become the focus of social attention due to the rapid pace of aging [24]. However, a significant gap remains in research concerning the balance between the care demands of older people and the services provided. Some research on care services primarily emphasizes medical care for frail, empty-nest, dementia-affected, and disabled older people [25–28]. With accelerated economic development and urbanization, the lifestyles and care service demands of older people have evolved. Simultaneously, the change of cultural concepts has promoted the pursuit of quality and individuation of care services for older people. Therefore, it is imperative to explore more effective care service models that address the challenges posed by the diverse demands of older people.

To address these limitations, this study constructs an overall evaluation system for care services according to the demands of older people. The innovations of our work are highlighted as follows: 1) According to the demands of older people, the critical evaluation indicators and their weights of care facilities are obtained; 2) For the older people with clear demands, we will support selecting care facilities according to their demands; 3) For the older people with non-clear demands, the orientation of care facilities is provided; 4) Care facilities are positioned and divided into four types to meet different types of older people. The study’s findings will advance the field of care services to provide better support for healthy aging.

## Methods

### Data Collection

With the improvement of medical technology and living standards, the number of older people is increasing. Taking Jinjiang District of Chengdu as an example, the population over 60 years old accounted for 20.86 percent of the total population by the end of 2022. Considering the changes in the social structure, the survey objects of this study include people aged 50 to 59 in addition to those over 60 years old. To ensure the success of the investigation, this study chooses random sampling to conduct offline and online surveys. Participants were randomly selected from the identified survey locations and online channels. The offline survey was conducted primarily in two communities with a higher proportion of older people (Jinguanyi and Jiaozi Community), a city park (Wangjiang Tower Park), and a senior center (Jiaozi Community). The online survey was conducted using Wenjuanxing (a professional online survey platform in China). Participants were recruited through community WeChat groups and local community announcements. All participants were informed of the study’s purpose, voluntary nature, and anonymity.

The questionnaire included the respondents’ demographics (age, gender, and education level), family structures (living arrangement and number of children), financial status (monthly income), demands and perceptions for care services. Three rounds of data collection were conducted in 2023 in March, July, and September. A total of 26 questionnaires were distributed in the first round of the survey, which served as a preliminary survey to improve the questionnaire design but were excluded from the results analysis. A total of 267 questionnaires were collected in the second and third rounds, including 223 qualified questionnaires. Participants were primarily younger older people (under 70 years old). The proportion of women was slightly higher than men, at 56.95%. Most participants had a secondary school education, representing 50.22% of the sample. Moreover, most of the participants’ monthly income is between 3,001 and 4000 CNY, accounting for 45.74%. The most common family structure involved one surviving child, with 35.43% of participants, and most participants lived either with their mate or with their children. Detailed demographic characteristics can be found in the Supplementary Appendix (Supplementary Table S1). Meanwhile, five experts $e_{r}$ with backgrounds in nursing, gerontology, and business management were employed to evaluate 10 care facilities $x_{i}$ .

### Variables

The outcome variable was the degree of care service demand among older people. Participants assessed their overall care service demands using a three-point scale: 1 (low), 2 (general), and 3 (high). The condition variables are the degrees of demands for the six types of care services provided by facilities, including medical services $c_{1}$ , psychological counseling $c_{2}$ , social activities $c_{3}$ , living conditions $c_{4}$ , diet conditions $c_{5}$ , and nursing services $c_{6}$ . Responses are provided on a five-point Likert scale (1 = “very not needed” to 5 = “very needed”). Related contents are shown in the Supplementary Materials.

To ensure the reliability of the measurements, we conducted reliability and validity analysis for the six condition variables. The overall Cronbach’s α for the scale was 0.852, and the Cronbach’s α for each condition variable ranged from 0.757 to 0.841, indicating good internal consistency [29]. Confirmatory factor analysis was used to check the validity of the measured items. The results revealed that all the factor loadings were above 0.701, demonstrating significant relationships between items and their latent constructs. Further, all condition variables exhibited average variance extraction (AVE) values greater than 0.587 and composite reliability (CR) values greater than 0.721, showing good construct reliability [30]. These results confirm the reliability of the scales. The detailed results can be found in the Supplementary Appendix (Supplementary Table S2).

### Demands Analysis

Fuzzy-set qualitative comparison analysis (fsQCA) is a variant of qualitative comparison analysis that describes the paths that produce the intended results, emphasizing combinations superior to single contributions [31]. Moreover, it emphasizes case-oriented comparative analysis from a holistic perspective [32]. Compared to other qualitative methods, fsQCA can explore complex combinations of multiple care service demands, identify configurations of different services, and work with small to medium sample sizes. Hence, it is suitable to address multi-factor complexity problems.

This study uses the direct calibration method to convert each variable into a fuzzy set. Three calibration anchors are determined using 90%, 50%, and 10% of the result and condition variable scores, corresponding to complete membership, cross point, and non-membership. Since all the consistency values are smaller than 0.9 in necessity analysis results, it indicates that any condition variable does not constitute the necessity condition of the outcome variable [33]. The truth table has 64 different combinations of conditions. The outcome variable is encoded as 1 when raw consistency is greater than 0.8 and PRI consistency is greater than 0.7 and both are met simultaneously; otherwise, it is encoded as 0. The configuration analysis aims to show specific results produced by various combinations of condition variables. After eliminating situations that do not reach the frequency and consistency threshold, the complex, intermediate, and parsimonious solutions are generated by the results of configuration analysis. First, the configuration of condition variables is classified using the intermediate solution. Next, the parsimonious solution is used to distinguish between the core and edge conditions for the condition variables. Table 1 shows the results of the configuration.

**TABLE 1:** Results of configuration (China, 2023).

Variables	Paths			
	1	2	3	4
Medical services $c_{1}$		●	●	●
Psychological counseling $c_{2}$	●	●	●	⊗
Social activities $c_{3}$	●	●		⊗
Living conditions $c_{4}$	●		●	⊗
Diet conditions $c_{5}$	●	⊗	●	⊗
Nursing services $c_{6}$	⊗	●	●	•
Consistency	0.907	0.887	0.880	0.857
Raw coverage	0.227	0.216	0.281	0.224
Unique coverage	0.054	0.044	0.071	0.087
Consistency of solution	0.860			
Coverage of solution	0.490			

Notes: Intermediate solution and parsimonious solution. The symbol ● means that the core condition variable exists and is assigned a score of 5; The symbol • means that the edge condition variable exists and is assigned a score of 4; Blank space means that the condition variable is optional and is assigned a score of 3; The symbol ⨰ means that the edge condition variable does not exist and is assigned a score of 2; The symbol ⊗ means that the core condition variable does not exist and is assigned a score of 1.

According to Table 1, there are four paths for the high demands, and the consistency of the solution is greater than 0.8. It reflects that all paths are sufficient conditions for forming high demands.

### Assessment and Positioning of Care Facilities

As a flexible tool to express people’s linguistic information, probabilistic linguistic term sets (PLTSs) [34] and some extension theories have been widely used in various practical problems [35, 36]. To effectively capture the uncertainty and vagueness in older people’s linguistic expressions and to describe their preferences, this study adopts the PLTSs [34, 37, 38]. PLTSs allow for expressing evaluations with multiple linguistic terms along with their associated probabilities.

For example, let $\left\{s_{-2}\left(very\;poor\right),s_{-1}\left(poor\right),s_{0}\left(general\right),s_{1}\left(good\right),s_{2}=\left(very\;good\right)\right\}$ be the linguistic term set used to evaluate facilities. Suppose that the PLTS for the facility $x_{1}$ in respect of the attribute $c_{1}$ is expressed as $L_{11}\left(p\right)=\left\{s_{-1}\left(0.4\right),s_{0}\left(0.6\right)\right\}$ , then it means that the facility $x_{1}$ performs “poor” in the attribute $c_{1}$ with a probability of 0.4 and “general” with a probability of 0.6. Moreover, the belief interval interpretation $BI\left(L_{11}\left(p\right)\right)=\langle \left[{B_{11}}^{-},{P_{11}}^{-}\right],\left[{B_{11}}^{+},{P_{11}}^{+}\right]\rangle =\langle \left[0.4,1\right],\left[0,0.6\right]\rangle$ indicates that the belief interval for the facility $x_{1}$ to perform “poor” in the attribute $c_{1}$ is $\left[0.4,1\right]$ , and the belief interval for it to perform “good” is $\left[0,0.6\right]$ . The use of belief interval interpretation allows for a more flexible and nuanced representation of the probabilistic linguistic information. For convenience, the belief interval interpretation for each decision matrix is expressed as $R^{r}=BI{\left(L_{ij}^{r}\left(p\right)\right)}_{10\times 6}$ .

Older people who choose care facilities can generally be divided into two groups. The first group consists of clear and specific demands, often prioritizing certain services offered by care facilities. The second group, however, may not have clearly defined demands and instead seek care facilities with strong overall capabilities. Therefore, two recommendation methods are established in this study.

#### Recommendation Method for Older People with Clear Demands

Step 1 Calculate the subjective weights of criteria (variables). Symbols reflect the presence status and significance of attributes within paths. The original coverage indicates the importance of each path in the configuration. Therefore, multiplying them together and performing the following operations can reveal the weight of attributes in the configuration. Let $S_{jk}$ be the score of the j−th variable in Table 1 for the k−th path and $RC_{k}$ be the raw coverage of the k−th path. Then the subjective weight $\omega_{j}$ of the criterion $c_{j}$ can be obtained by $\omega_{j}=\sum_{k=1}^{4}S_{jk}RC_{k}/\sum_{j=1}^{6}\sum_{k=1}^{4}S_{jk}RC_{k}$ according to the configuration analysis results.

Step 2 Get the similarity matrix. This step measures the consistency of expert opinions by calculating similarity between their PLTSs. Let $L_{ij}^{a}\left(p\right)$ and $L_{ij}^{b}\left(p\right)$ be PLTSs of the criterion $c_{j}$ corresponding to experts $e_{a}$ and $e_{b}$ for the facility $x_{i}$ , then the similarity measure between $e_{a}$ and $e_{b}$ is defined as Equation 1.

$$SI\left(e_{a},e_{b}\right)=1-10\times \omega_{j}\sum_{i=1}^{10}\sum_{j=1}^{6}D\left(e_{a},e_{b}\right)$$ (1)

where $D\left(e_{a},e_{b}\right)=\frac{1}{2}\max\left(\left|{B_{ij}}^{a-}-{B_{ij}}^{b-}\right|+\left|{B_{ij}}^{a+}-{B_{ij}}^{b+}\right|,\left|{P_{ij}}^{a-}-{P_{ij}}^{b-}\right|+\left|{P_{ij}}^{a+}-{P_{ij}}^{b+}\right|\right)$ . Then the similarity matrix can be obtained.

Step 3 Obtain the weights of experts according to Equation 2. Expert weights are derived based on their consistency, where more consistent experts receive higher weights. Sum of each row of the similarity matrix is denoted as $\Phi_{r}$ . According to the above definitions, the weight of the expert $e_{r}$ is expressed as

$$\sigma_{r}=\frac{\Phi_{r}-1}{\sum_{r=1}^{5}\Phi_{r}-5}$$ (2)

Step 4 Get the entropy measure, and the objective weights of criteria. Entropy for each criterion is calculated to measure assessment uncertainty, where higher entropy indicates greater importance in differentiating facility performance. Let $L_{ij}^{r}\left(p\right)$ be PLTS of the criterion $c_{j}$ corresponding to the expert $e_{r}$ for the facility $x_{i}$ , then the entropy measure for $c_{j}$ is defined as Equation 3.

$$E_{j}=\frac{\sigma_{r}}{10}\sum_{i=1}^{10}\sum_{r=1}^{5}D\left(BI\left(L_{ij}^{r}\left(p\right)\right),\Psi_{\max}\right)$$ (3)

where $\Psi_{\max}=\langle \left[0,0\right],\left[1,1\right]\rangle$ . Then the objective weight of the criterion $c_{j}$ can be obtained by Equation 4.

$$\varpi_{j}=\frac{1-E_{j}}{6-\sum_{j=1}^{6}E_{j}}$$ (4)

Step 5 Calculate the comprehensive weights of criteria. This step maximizes the divergence of assessed facility values to identify key discriminating criteria for weighting. The evaluation matrix can be defined as $Α={\left(v_{ij}\right)}_{10\times 6}$ , $v_{ij}=\sigma_{r}\sum_{r=1}^{5}D\left(BI\left(L_{ij}^{r}\left(p\right)\right),\Psi_{\min}\right)$ , and $\Psi_{\min}=\langle \left[1,1\right],\left[0,0\right]\rangle$ . Next, with the goal of maximizing the deviation of facilities’ assessed values, the following programming model is constructed, see Equation 5. And the comprehensive weight $w_{j}$ of the criterion $c_{j}$ can be obtained by solving it.

$$\begin{array}{l} \max\Delta =\sum_{i=1}^{10}\left|V_{i}-\overset{-}{V}\right|\\ s.t.\left\{\begin{array}{l} V=W^{c}{Α}^{T}\\ \overset{-}{V}=\frac{1}{10}\sum_{i=1}^{10}V_{i}\\ \sum_{j=1}^{6}w_{j}=1\\ 0\leq w_{j}\leq 1\\ \min\left(\omega_{j},\varpi_{j}\right)\leq w_{j}\leq \max\left(\omega_{j},\varpi_{j}\right) \end{array}\right. \end{array}$$ (5)

where $W^{c}=\left(w_{1},w_{2},\ldots ,w_{6}\right)$ , and $V_{i}$ is the assessed value of the facility $x_{i}$ .

Step 6 Calculate the score values of facilities. Facility performance for each criterion is quantified using the concept of dominance, where positive scores indicate superior performance. The dominance degree of each facility $x_{i}$ over the facility $x_{q}\left(q\neq i\right)$ with respect to the criterion $c_{j}$ is defined as Equation 6.

$$\vartheta_{j}\left(x_{i},x_{q}\right)=\left\{\begin{array}{l} \sqrt{\left(v_{ij}-v_{qj}\right)w_{j}^{\prime }/\sum_{j=1}^{6}w_{j}^{\prime }},if\;v_{ij}-v_{qj}>0\\ 0,if\;v_{ij}-v_{qj}=0\\ -\frac{1}{\theta }\sqrt{\left(v_{qj}-v_{ij}\right)\sum_{j=1}^{6}w_{j}^{\prime }/w_{j}^{\prime }},if\;v_{ij}-v_{qj}<0 \end{array}\right.$$ (6)

where $w_{j}^{\prime }=w_{j}/\max w_{j}$ . The parameter $\theta \left(\theta >0\right)$ is the attenuation coefficient in the face of loss, the smaller it is, the higher the degree of loss avoidance. Then the score value of the facility $x_{i}$ with respect to the criterion $c_{j}$ can be obtained by $\phi_{j}\left(x_{i}\right)=\sum_{q=1,q\neq i}^{10}\vartheta_{j}\left(x_{i},x_{q}\right)$ .

#### Recommendation Method for Older People with Non-Clear Demands

The competitive advantage and the potential risk are two key indicators for assessing the performance and prospects of care facilities. Competitive advantage degree is used to evaluate the advantage of facilities relative to competitors. Facilities with a high degree of competitive advantage may perform well in certain key aspects, and are often able to attract more older people. They will maintain a high occupancy rate and gain a good reputation. Potential risk degree is used to assess the potential risks and uncertainties faced by facilities. Potential risks may come from many aspects, including market competition, personnel management, financial problems, etc. A high potential risk may mean more challenges for the facility, which may affect the facility’s profitability and customer satisfaction.

Steps 1–6 are the same as recommendation method for older people with clear demands.

Step 7 Obtain the competitive advantage degree and potential risk degree. Competitive advantage and potential risk are calculated using a comparison matrix that captures relative dominance and an uncertainty factor. The overall dominance degree of each facility $x_{i}$ over the facility $x_{q}\left(q\neq i\right)$ can be obtained by $\varphi \left(x_{i},x_{q}\right)=\sum_{j=1}^{6}\vartheta_{j}\left(x_{i},x_{q}\right)$ . The elements in comparison matrix can be calculated by Equation 7.

$$\upsilon_{iq}=\left\{\begin{array}{l} e^{\lambda \frac{\varphi \left(x_{i},x_{q}\right)-\varphi^{-}\left(x_{i}\right)}{\varphi^{+}\left(x_{i}\right)-\varphi^{-}\left(x_{i}\right)}},\varphi \left(x_{i},x_{q}\right)\neq 0\\ 0,\varphi \left(x_{i},x_{q}\right)=0 \end{array}\right.$$ (7)

where $\varphi^{+}\left(x_{i}\right)=\max\left\{\varphi \left(x_{i},x_{q}\right)\left|q\neq i\right.\right\},\varphi^{-}\left(x_{i}\right)=\min\left\{\varphi \left(x_{i},x_{q}\right)\left|q\neq i\right.\right\}$ . The parameter $\lambda \left(\lambda >0\right)$ is the uncertainty factor, the higher the value of it, the higher the degree of influence on competitive advantage and potential risk. The competitive advantage degree $\Omega_{i}$ and potential risk degree ${℧}_{i}$ of the facility $x_{i}$ are defined as the row sum and column sum of the comparison matrix, respectively.

Step 8 Draw the positioning of care facilities.

**FIGURE 1:**
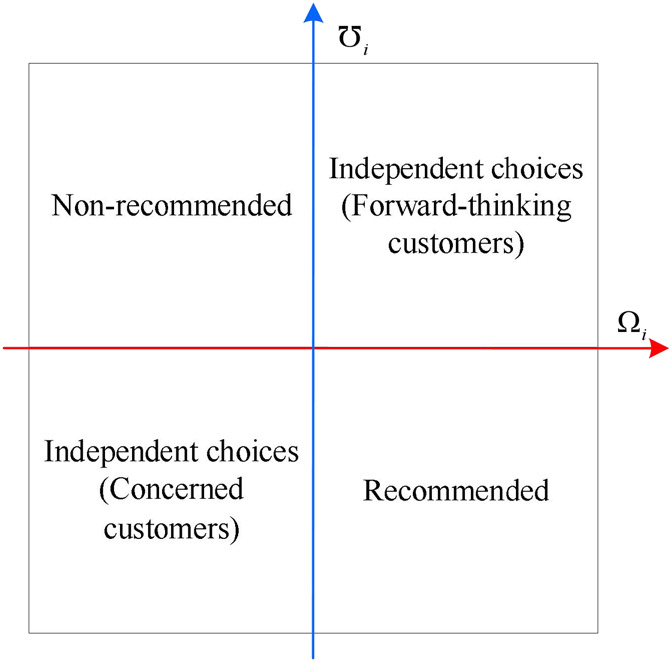
The positioning of different types of care facilities (China, 2023).

In this study, a novel approach is used to provide a new perspective on the selection of care facilities by constructing a coordinate system of competitive advantage degree and potential risk degree. By dividing the four quadrants in the coordinate system, we can position different types of care facilities more clearly and thus provide recommendation strategies for different types of customers. The details can be found in Figure 1. The coordinate point composed of average competitive advantage degree and average potential risk degree is chosen as the origin.

The first quadrant represents care facilities with high levels of both competitive advantage and potential risk. Facilities in this quadrant are recommended to forward-thinking customers who are typically open to risk and willing to explore new opportunities and experiences.

The second quadrant represents facilities with lower levels of competitive advantage and higher levels of potential risk. In this case, customers are less likely to be advised to choose these facilities. Although there may be some attractive factors, low competitive advantage can affect customers’ experience.

In the third quadrant, both competitive advantage and potential risk are lower. These are suitable facilities to recommend to concerned customers who are more focused on stability and avoiding potential risks. While these facilities may lack prominence, they may be better at providing stability and reliable services.

The fourth quadrant presents facilities with higher competitive advantage but lower potential risk. In this case, customers are encouraged to prioritize these facilities. This is because these facilities provide satisfactory services while reducing the possibility of exposure to potential risks, allowing customers to achieve a sustained sense of satisfaction.

## Results

In this study, the comprehensive weights of criteria can be obtained as $w_{1}=0.18,w_{2}=0.18,w_{3}=$ $0.16,w_{4}=0.16,w_{5}=0.14,w_{6}=0.18$ . The score values of facilities can be found in Table 2.

**TABLE 2:** The score values of care facilities (China, 2023).

	$c_{1}$	$c_{2}$	$c_{3}$	$c_{4}$	$c_{5}$	$c_{6}$
$x_{1}$	0.6751	1.6683	−1.8618	−3.7218	−0.8191	−4.3252
$x_{2}$	−3.9796	−5.8574	0.0706	−2.3796	−0.6388	−4.8671
$x_{3}$	−4.9910	−2.6655	−11.3013	−1.0380	−6.4939	−2.3315
$x_{4}$	−0.3018	−2.3162	1.7691	−3.4762	−3.5717	−5.9636
$x_{5}$	1.1699	−10.5527	−6.1940	1.1328	−0.0049	1.7785
$x_{6}$	0.1949	−2.2503	−1.9104	−5.2230	−7.6624	−3.8080
$x_{7}$	−1.2692	−7.0268	−6.7460	−11.2122	−3.2070	−0.5920
$x_{8}$	−9.0855	0.0246	0.8943	−1.7102	−2.6836	0.5424
$x_{9}$	−5.9565	−4.3149	−7.7865	0.1533	1.5847	−5.5668
$x_{10}$	−2.0654	1.3197	−5.1390	0.5675	−10.9828	−1.2610

Therefore, care facilities can be recommended according to the results above. For example, the ranking of medical services $c_{1}$ is $x_{5}≻x_{1}≻x_{6}≻x_{4}≻x_{7}≻x_{10}≻x_{2}≻x_{3}≻x_{9}≻x_{8}$ , the care facility $x_{5}$ is preferentially recommended for older people with clear medical services demands. In addition, older people can choose freely according to the score values under different criteria of care facilities.

To verify the robustness of the methods, different values of the parameter θ are set to 0.2, 0.4, 0.6, 0.8, 1, 1.5, 2, 5, and 10 respectively. The results are shown in Table 3.

**TABLE 3:** Ranking of care facilities at parameter θ (China, 2023).

Criteria	Ranking
$c_{1}$	$x_{5}≻x_{1}≻x_{6}≻x_{4}≻x_{7}≻x_{10}≻x_{2}≻x_{3}≻x_{9}≻x_{8}$
$c_{2}$	$x_{1}≻x_{10}≻x_{8}≻x_{6}≻x_{4}≻x_{3}≻x_{9}≻x_{2}≻x_{7}≻x_{5}$
$c_{3}$	$x_{4}≻x_{8}≻x_{2}≻x_{1}≻x_{6}≻x_{10}≻x_{5}≻x_{7}≻x_{9}≻x_{3}$
$c_{4}$	$x_{5}≻x_{10}≻x_{9}≻x_{3}≻x_{8}≻x_{2}≻x_{4}≻x_{1}≻x_{6}≻x_{7}$
$c_{5}$	$x_{9}≻x_{5}≻x_{2}≻x_{1}≻x_{8}≻x_{7}≻x_{4}≻x_{3}≻x_{5}≻x_{10}$
$c_{6}$	$x_{5}≻x_{8}≻x_{7}≻x_{10}≻x_{3}≻x_{6}≻x_{1}≻x_{2}≻x_{9}≻x_{4}$

The ranking is constant for different values of the parameter θ , which fully proves the robustness of the proposed method. It can provide useful information for older people and their family members to choose care facilities.

**FIGURE 2:**
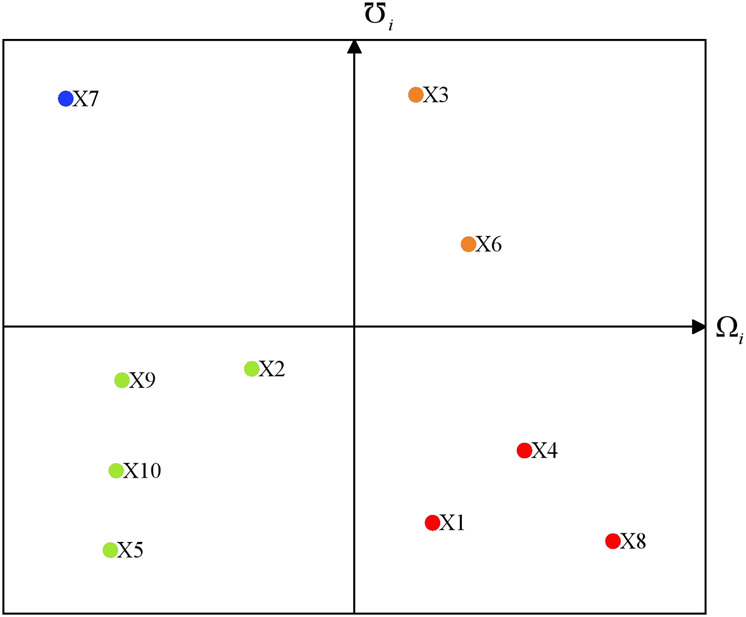
The positioning of care facilities in case study (China, 2023).

The competitive advantage degree and potential risk degree can be obtained, and the positioning of care facilities can be visually observed in Figure 2.

Care facilities $x_{1}$ , $x_{4}$ , and $x_{8}$ are preferentially recommend to older people who do not have clear demands. Care facilities $x_{3}$ and $x_{6}$ may attract forward-thinking customers. Care facilities $x_{2}$ , $x_{5}$ , $x_{9}$ , and $x_{10}$ are suitable for concerned customers. However, the care facility $x_{7}$ is not recommended for older people. We encourage customers to choose a care facility based on their characteristics and actual circumstances.

**FIGURE 3:**
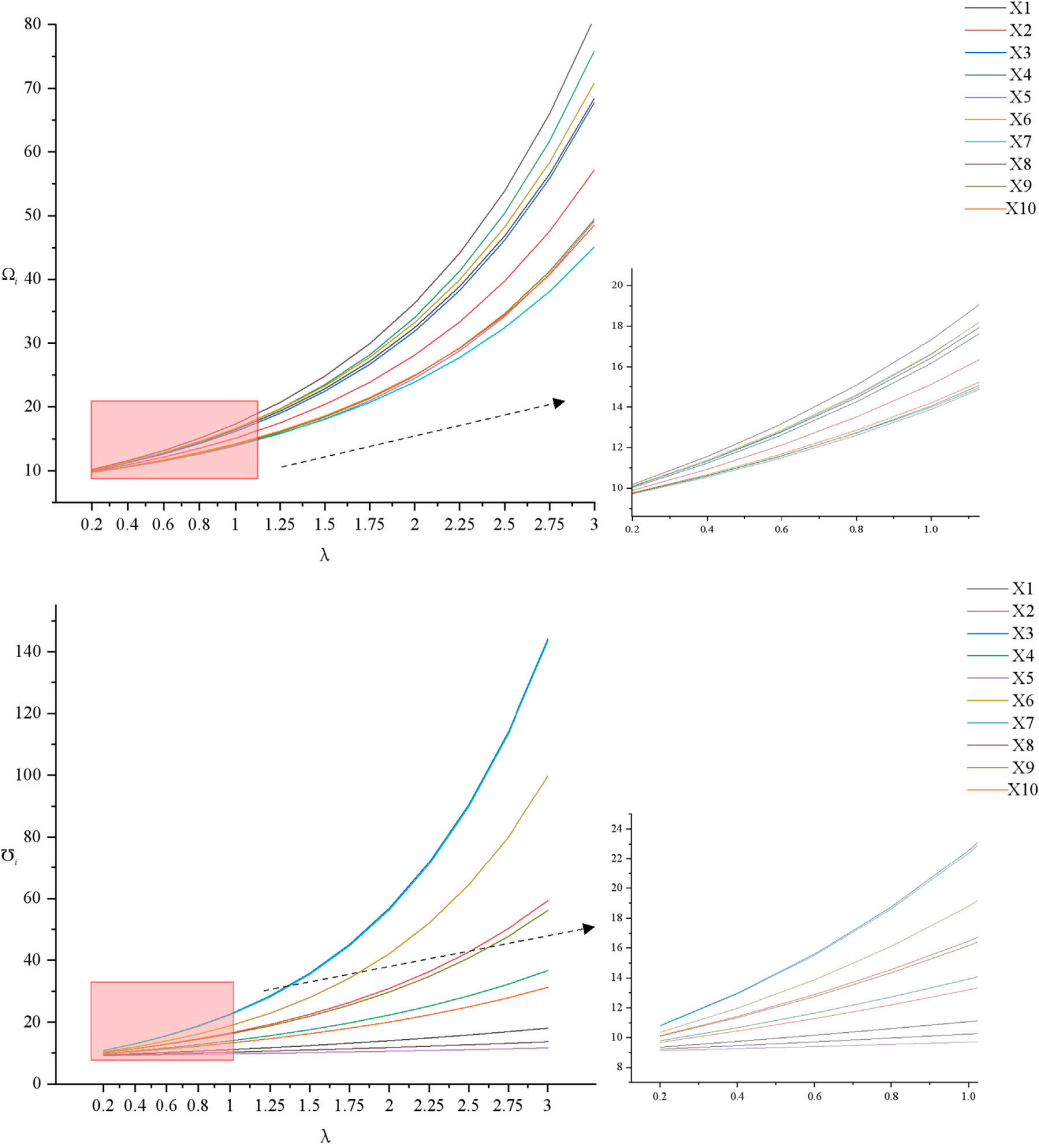
The sensitivity analysis of parameter λ (China, 2023).

Then the sensitivity of the parameter λ is analysed to prove the robustness of the second recommendation method. The results are shown in Figure 3.

We can find that with the increase of the parameter λ , both the difference of competitive advantage degrees and potential risk degrees are gradually obvious. But it basically does not affect the ranking. Hence, the two recommendation methods proposed in this study have good performance.

## Discussion

### Revelation for the Demands of Older People

This study identified four distinct paths of factors contributing to the high demand for care services, highlighting the diverse demands of older people. These results align with prior studies [15, 39] that have also demonstrated the heterogeneity of care demands among older people. However, our study goes further by pinpointing specific combinations of care services that lead to higher care demands through the application of fsQCA. However, most of the existing studies adopt regression-based methods, focusing on analyzing the isolated effects of individual variables, while ignoring the synergistic effects of variable combinations [40, 41].

The different paths reveal the differences and characteristics among the demands of older people. Path 1 shows that the older people have low demands for nursing services, which implies that they maintain a relatively independent lifestyle without relying heavily on conventional healthcare services. The remaining 3 paths show the demands for medical and nursing services, and there are differences in the demands for other services. Specifically, the older people in Path 2 may have a higher tendency toward extroversion, psychological counseling, and social activities. They expect to gain psychological pleasure and social contact in their later years [42]. Therefore, a range of social activities can be provided by care facilities to satisfy their demands. On the contrary, the older people in Path 3 do not expect much social activity. In addition to satisfactory living and diet conditions, they also demand psychological satisfaction. Their mental health issues should be paid attention to by facilities [43]. Path 4 shows that the older people strongly demand medical and nursing services. They demand more healthcare services and may suffer from chronic diseases or reduced physical mobility.

Overall, these findings reveal a high overall demand for medical services, psychological counselling, and nursing services. This is consistent with studies emphasizing the importance of these services for the wellbeing of older people [44–46]. Diversified demands analysis can help care facilities provide personalized services more responsive to older people. Moreover, such analysis provides policymakers with a valuable reference for developing targeted care service strategies [47]. To effectively address these diverse demands, governments should move beyond a singular focus on medical care and allocate resources toward a more comprehensive and responsive approach. Specifically, policymakers should formulate policies based on the results of demand analysis.

### Revelation for Assessment of Care Facilities

This study aims to provide recommendation methods for older people with different demands to choose care facilities. For older people with clear demands, care facilities can be selected according to the first recommendation method. For older people with non-clear demands, facilities can be selected by the second recommendation method. Both methods can meet the various demands of older people and offer more suitable choices for older people. Besides, a greater understanding of demands can help care facilities improve service quality more effectively [48]. Additionally, governments can rationally allocate care services resources and supervise according to evaluation and positioning results to promote the sustainable development of the care industry.

Moreover, the sensitivity analysis shows that our methods are suitable for different situations. Lower θ indicates that the method is more sensitive to differences between different facilities. Conversely, higher the value of λ , the more likely it is to highlight the differences in strengths and weaknesses of these facilities. Therefore, setting reasonable values for the parameters can balance the differentiation and fairness of evaluation results. Lower values for parameter θ and higher values for parameter λ may enable decision makers to see more clearly the differences of facilities. This also makes it easier for older people to choose and helps optimize resource allocation and service improvements. Otherwise, the difference may be suppressed, affecting the accuracy of the evaluation.

### Limitations

There are several limitations in this study. First, the demands of older people surveyed were cross-sectional, which was limited by its self-reported nature. It only selected six types of care services provided by care facilities and might not reflect long-term changes in the demands of older people. Second, this study only included 223 older people and 10 care facilities in Chengdu, China, and the generalizability of the results was limited by geography and sample size. Third, the findings may be influenced by the specific cultural, economic, and social contexts of China. Therefore, there are limitations to the applicability of the results to other regions or countries with different backgrounds. In future research, it is possible to obtain a more diversified demand for older people by expanding the sample and scope of the survey objects. We may also pay attention to the dynamic changes in the quality of care facilities to provide dynamic recommendations for older people.

### Conclusion

Through demands analysis, it was found that there were four paths leading to high care services demands of older people. The analysis revealed that medical services, psychological counseling, and nursing services exerted the most significant influence on demand, while diet conditions had the least impact. Furthermore, targeted recommendation methods were provided for older people with different demands, and the robustness of these methods was demonstrated through sensitivity analysis. This study provides valuable insights for older people, care facilities, and governments, enabling them to better address care demands and promote improved living environments.
