# Supplementary material for: Balancing the Demands of Older People and Care Services of Healthy Aging: Assessment and Positioning of Care Facilities
Source: Int J Public Health. 2025 Jun 30;70:1607249. doi: 10.3389/ijph.2025.1607249 (PMC12256299; doi:10.3389/ijph.2025.1607249)
Supplement: Supplementary file 1 [file Table1.docx]

**Table S1.** Demographics of the participants (China, 2023).

|  | *N* | Pencent (%) |
| --- | --- | --- |
| Age |  |  |
| 50-59 | 66 | 29.60 |
| 60-69 | 77 | 34.53 |
| 70-79 | 52 | 23.32 |
| 80-89 | 28 | 12.56 |
| Gender |  |  |
| Men | 96 | 43.05 |
| Women | 127 | 56.95 |
| Education level |  |  |
| Primary school and below | 74 | 33.18 |
| Secondary school | 112 | 50.22 |
| College | 19 | 8.52 |
| Bachelor | 11 | 4.93 |
| Master or above | 7 | 3.14 |
| Income (CNY) |  |  |
| ≤2000 | 43 | 19.28 |
| 2001-3000 | 57 | 25.56 |
| 3001-4000 | 102 | 45.74 |
| ＞4000 | 21 | 9.42 |
| Surviving children |  |  |
| 0 | 13 | 5.83 |
| 1 | 79 | 35.43 |
| 2 | 67 | 30.04 |
| 3 | 46 | 20.63 |
| ≥4 | 18 | 8.07 |
| Living |  |  |
| Alone | 24 | 10.76 |
| With mate | 63 | 28.25 |
| With children | 73 | 32.74 |
| With mate and children | 40 | 17.94 |
| In care facility | 23 | 10.31 |
