# Supplementary material for: Balancing the Demands of Older People and Care Services of Healthy Aging: Assessment and Positioning of Care Facilities
Source: Int J Public Health. 2025 Jun 30;70:1607249. doi: 10.3389/ijph.2025.1607249 (PMC12256299; doi:10.3389/ijph.2025.1607249)
Supplement: Supplementary file 2 [file Table2.docx]

**Table S2.** Results of reliability and validity analysis (China, 2023).

| Variable | | Cronbach’s α | | Loading | AVE | CR |
| --- | --- | --- | --- | --- | --- | --- |
| Medical services | MS1 | 0.823 | 0.852 | 0.825 | 0.702 | 0.843 |
|  | MS2 |  |  | 0.827 |  |  |
| Psychological counseling | PC1 | 0.781 |  | 0.792 | 0.685 | 0.788 |
|  | PC2 |  |  | 0.768 |  |  |
| Social activities | SA1 | 0.757 |  | 0.745 | 0.612 | 0.752 |
|  | SA2 |  |  | 0.712 |  |  |
| Living conditions | LC1 | 0.803 |  | 0.812 | 0.665 | 0.801 |
|  | LC2 |  |  | 0.789 |  |  |
| Diet conditions | DC1 | 0.766 |  | 0.734 | 0.587 | 0.721 |
|  | DC2 |  |  | 0.701 |  |  |
| Nursing services | NS1 | 0.841 |  | 0.845 | 0.723 | 0.856 |
|  | NS2 |  |  | 0.867 |  |  |
