# Supplementary material for: Balancing the Demands of Older People and Care Services of Healthy Aging: Assessment and Positioning of Care Facilities
Source: Int J Public Health. 2025 Jun 30;70:1607249. doi: 10.3389/ijph.2025.1607249 (PMC12256299; doi:10.3389/ijph.2025.1607249)
Supplement: Supplementary file 3 [file DataSheet2.docx]

**The Elderly Demands Questionnaire**

1. Which of the following service standards are you concerned about? (Multiple choices)

□ Medical services

□ Psychological counseling

□ Social activities

□ Living conditions

□ Diet conditions

□ Nursing services

□ Other _____________

2. What is your degree of demand for elderly care services? (Single choice)

□ Low

□ General

□ High

1. What is your degree of demand for simple medical services? (Single option)

□Very not needed

□Relatively not needed

□General

□Relatively needed

□Very needed

4. What is your degree of demand for professional medical services? (Single option)

□Very not needed

□Relatively not needed

□General

□Relatively needed

□Very needed

5. What is your degree of demand for mental health counseling? (Single option)

□Very not needed

□Relatively not needed

□General

□Relatively needed

□Very needed

6. What is your degree of demand for stress management? (Single option)

□Very not needed

□Relatively not needed

□General

□Relatively needed

□Very needed

7. What is your degree of demand for recreational activities? (Single option)

□Very not needed

□Relatively not needed

□General

□Relatively needed

□Very needed

8. What is your degree of demand for cultural and sports activities? (Single option)

□Very not needed

□Relatively not needed

□General

□Relatively needed

□Very needed

9. What is your degree of demand to live comfortably? (Single option)

□Very not needed

□Relatively not needed

□General

□Relatively needed

□Very needed

10. What is your degree of demand for safe living conditions? (Single option)

□Very not needed

□Relatively not needed

□General

□Relatively needed

□Very needed

11. What is your degree of demand for nutritional requirements? (Single option)

□Very not needed

□Relatively not needed

□General

□Relatively needed

□Very needed

12. What is your degree of demand for delicious food? (Single option)

□Very not needed

□Relatively not needed

□General

□Relatively needed

□Very needed

13. What is your degree of demand for health assessment? (Single option)

□Very not needed

□Relatively not needed

□General

□Relatively needed

□Very needed

14. What is your degree of demand for medication management? (Single option)

□Very not needed

□Relatively not needed

□General

□Relatively needed

□Very needed
